# Genetics in mainstream medicine: Finally within grasp to influence healthcare globally

**DOI:** 10.1002/mgg3.415

**Published:** 2018-05-28

**Authors:** Swaroop Aradhya, Robert L. Nussbaum

**Affiliations:** ^1^ Invitae San Francisco California; ^2^ Adjunct clinical associate professor Stanford University School of Medicine Stanford California; ^3^ Volunteer faculty University of California San Francisco San Francisco California

**Keywords:** clinical trial, diagnostic testing, exome, genetic screening, genomic databases, next‐generation sequencing, panels, precision medicine, therapy

## Abstract

A modern genomics ecosystem has emerged. This commentary describes recent trends in clinical genomics that enable its successful integration in mainstream medicine. The rapid expansion of clinical genomics will have a positive impact on the healthcare of individuals worldwide.

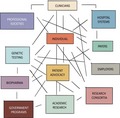

In January 1994, a *Time* magazine cover proclaimed “Genetics—the future is now.” Though it was prescient and certainly exciting to contemplate how genomics would transform healthcare and save lives, the proclamation was premature. A decade later, the completion of the Human Genome Project provided another occasion to expound on the merits and promises of genomic medicine. Despite this landmark scientific achievement and the subsequent exponential growth in our understanding of the genomic basis for human disease, the integration of genomics into healthcare has been slow because of the scarcity of access to appropriate genetic testing across all clinical specialties in which it matters. In this commentary, we describe the latest trends in clinical genomics as it relates to hereditary disorders and argue that we have finally reached a tipping point at which genomics has a realistic chance to radically transform medicine. This transformation will occur in key interrelated ways: by improving our understanding of the phenotypic spectrum of germline genetic disorders, enabling accurate molecular diagnoses in a greater number of individuals, connecting patients to therapies and clinical trials, and empowering individuals to comprehensively understand their genetic heritage, participate in patient advocacy, and consider informed proactive genetic screening for clinically actionable hereditary disorders. Together, these developments will stimulate a paradigm shift through the evidence‐based integration of genetics in various clinical specialties, thereby having a lasting impact on the healthcare of the global population.

Our optimism stems from a recent convergence of several essential components in a strengthening genomics ecosystem: rapidly expanding medical genetics knowledge, advances in genetic testing technologies and informatics solutions, the development of public genomics databases, large‐scale studies of clinical and research cohorts, and novel methods to improve phenotyping (Figure 1). Recent advances in DNA sequencing chemistries, microarrays, and bioinformatics have resulted in more expansive and affordable genetic testing (Boycott, Vanstone, Bulman, & MacKenzie, 2013). A crucial development in this evolution has been the switch from analog to digital genomic information: whole‐genome chromosomal microarrays (CMA) and next‐generation sequencing (NGS) are vastly scalable compared with traditional methods, and data from these new technologies are amenable to computational and statistical analyses that have yielded major discoveries and refined existing clinical genomics concepts.

**Figure 1:**
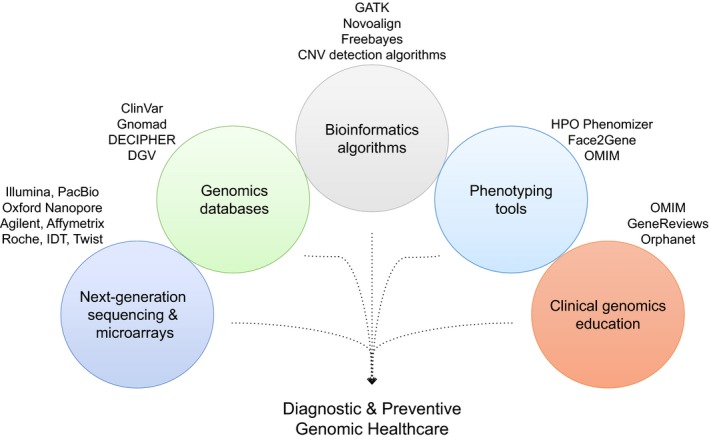
Convergence of components necessary for scalable and comprehensive genome analysis and interpretation, including representative examples of each

These advances in laboratory technologies and bioinformatics solutions have in turn encouraged the establishment and investigation of massive sets of genomic data from public and private efforts, dramatically enhancing our understanding of the variation that exists in both clinical cohorts and healthy individuals, and most importantly, across a variety of populations worldwide. Large‐scale initiatives such as OMIM, ClinGen, the 1,000 Genomes Project, gnomAD, DECIPHER, and the Database of Genomic Variants have been critical to the refinement of clinical genetic interpretations of sequence and copy number variants (Amberger & Hamosh, 2017; Firth et al., 2009; 1000 Genomes Project Consortium et al., 2015; Lek et al., 2016; MacDonald, Ziman, Yuen, Feuk, & Scherer, 2014; Rehm et al., 2015). These initiatives provide key tools and information with which to contextualize genomic data by comparing any individual with thousands of others whose genotypes and phenotypes have been assessed. A cliché is apt: Genomics is indeed a big data challenge.

The vast amount of data generated by CMA and NGS requires evidence‐based methods for analysis and clinical interpretation. Open data sharing among clinical laboratories during the last decade has been a major driving force behind improvements in the quality of clinical interpretation. Members of ClinGen and its predecessors have helped develop mechanisms and recommendations to promote the consistent interpretation of sequence and copy number variants (Richards et al., 2015; Riggs et al., 2012). These contributors are also curating the morbid genome and highlighting caveats for specific disorders to encourage better contextualized interpretation of clinical genetic results. Discordance in clinical interpretation has long been a difficult problem in clinical genomics, but databases such as ClinVar are successfully tackling this challenge and resolving interlaboratory differences in variant classifications (Landrum et al., 2016; Yang et al., 2017). Moving forward, clinical interpretation of germline genetic data will necessarily incorporate machine learning and other artificial intelligence (AI) tools to systematically mine databases like ClinVar or gnomAD, weigh the available evidence related to observed variants, and classify those variants on a scale of disease causation, using a Bayesian statistical framework (Tavtigian et al., 2018).

Today, clinical genomics as it relates to hereditary disorders is expanding its presence in mainstream medicine on two fronts: diagnostic testing and proactive screening. Relatively speaking, we are now ostensibly in a golden age for diagnostic genetic testing. At the time of this writing, the Genetics Test Registry listed 53,704 tests provided by 506 laboratories for 11,022 conditions associated with 16,420 genes (Rubinstein et al., 2013). Most genetic testing has shifted to NGS, providing high analytic sensitivity for a range of variant types that is broader than that typically associated with traditional methods such as Sanger sequencing. NGS enables the simultaneous identification of single‐nucleotide variants, small and large indels, exon‐level deletions and duplications, and even chromosomal copy number variants in a single, relatively low‐cost test (Lincoln et al., 2015; Retterer et al., 2015; Truty et al., 2018). Highly sensitive NGS‐based methods will soon replace CMA for chromosomal copy number variant analysis. Going forward, we can expect sophisticated bioinformatics algorithms to also enable the detection of triplet repeat expansions and methylation abnormalities in imprinting disorders (Liu, Zhang, Wang, Gu, & Wang, 2017; Tang et al., 2017). Eventually, all of these capabilities will be rolled into a single low‐cost whole‐genome sequencing test, thus ending a long journey to a reliable method that captures all clinically relevant variants in patients referred for testing for a suspected hereditary disorder (Figure 2).

**Figure 2:**
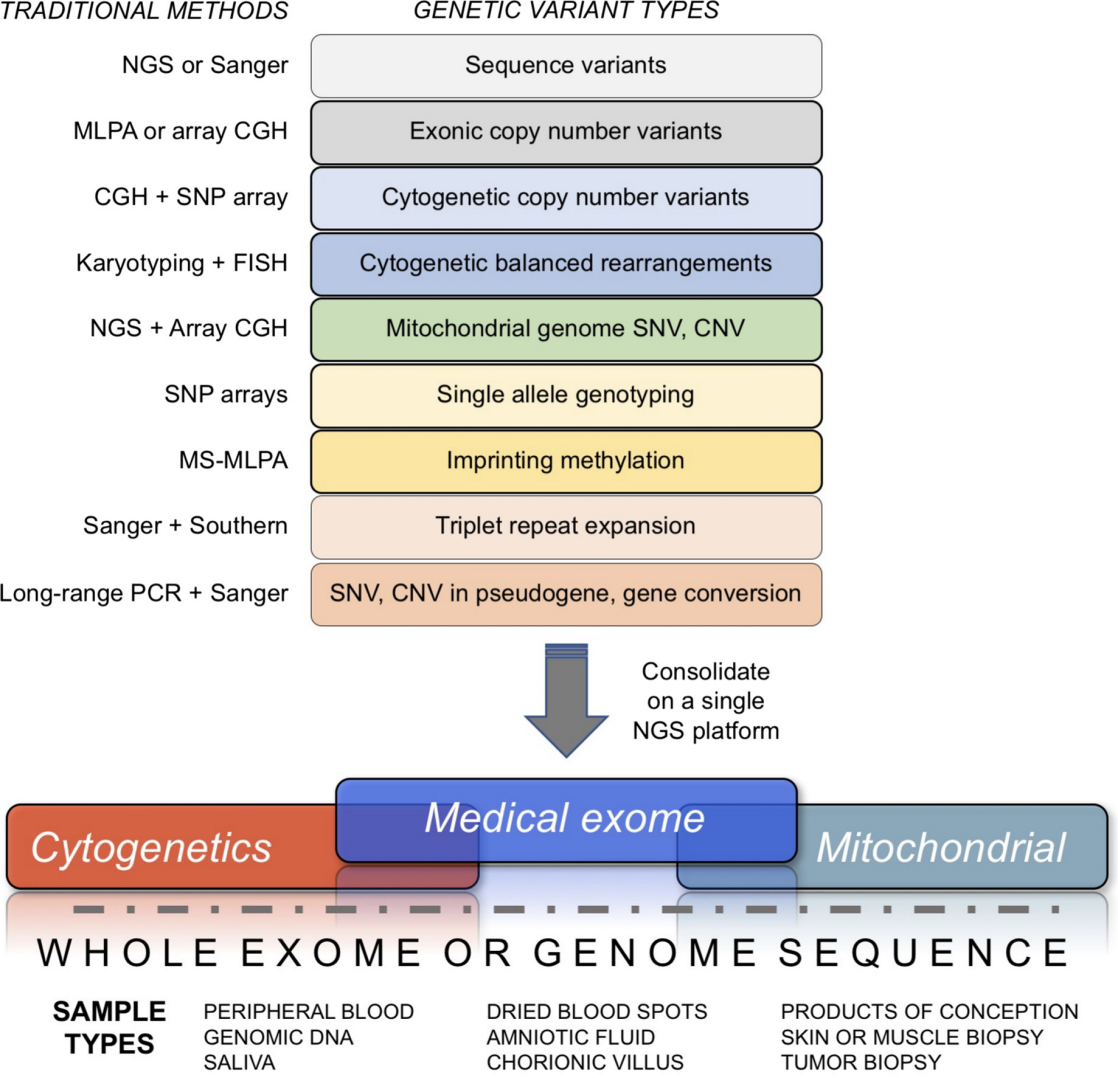
The imminent “one” germline genetic test. The consolidation of the detection of various clinically relevant variant types in the genome into a single test will eventually evolve into a whole‐exome or whole‐genome diagnostic method with high analytic sensitivity and specificity and will be performed on a range of sample types. This test will be able to identify chromosomal CNVs and balanced rearrangements as well as gene‐level SNVs and CNVs. In addition, the inclusion of mitochondrial genome sequencing will complement nuclear genome analysis. It will be important to ensure higher sequencing quality in the medically relevant portions of the genome (“medical exome”), which should be continually updated as novel disease genes are discovered. Genes with pseudogene copies and segmental duplications can be evaluated with NGS with optimized chemistry and bioinformatics. Triplet repeat expansions can be detected with long‐read NGS and other modifications, but identifying large expansions such as those in fragile X syndrome or myotonic dystrophy type II can be more challenging. CGH, comparative genomic hybridization; CNV, copy number variant; FISH, fluorescence in situ hybridization; MLPA, multiplex ligation‐dependent amplification; NGS, next‐generation sequencing; SNP, single‐nucleotide polymorphism; “SNV, single‐nucleotide variant

Within the next decade, the use of comprehensively assessed whole‐genome sequences for individuals with diagnosed or suspected germline disorders will likely become routine (and even coupled with other “omics” such as transcriptomics, proteomics, and metabolomics). However, at least two challenges will modulate the pace of this evolution: the high cost of whole‐genome sequencing and the limited understanding of the clinical relevance of noncoding regions of the genome. These challenges will surely be addressed as technologies improve and additional studies using whole‐genome sequencing are conducted around the world. In the meantime, targeted multigene NGS panels are having a significant impact on the accessibility of genetic testing owing to their versatility and low cost, and they are therefore increasing testing across clinical specialties and facilitating the deeper integration of genetics in medicine.

Among the advantages of such panels, superior analytic sensitivity and wide availability make them excellent options as first tests for many disorders that can be diagnosed in the clinic with a moderate to high degree of confidence. Moreover, these panels contain all known genes associated with a specific disorder and are updated periodically to add newly discovered genes. As a result, well‐designed panels have a useful diagnostic yield: a recent review reported a positive diagnostic yield of 10% to 60% (Truty et al., 2018). The higher yields were associated with disorders that clinicians can diagnose with high confidence and have few phenocopies (e.g., Duchenne muscular dystrophy and hyperphenylalaninemia).

Targeted NGS panels also yield relatively faster results, which can be particularly helpful in patients with disorders that have immediate clinical management implications—for example, prophylactic surgical options in breast or gastric cancers, life‐saving surveillance in colon cancer, the selection of replacement therapies in certain inborn errors of metabolism, appropriate choice of anti‐epileptic drugs based on genotype, and decisions to implant support devices in some hereditary cardiovascular disorders. Finally, targeted NGS panels can be paired with complementary tests to obtain fuller clinical assessments. For instance, pairing germline and tumor NGS panel testing in individuals with ongoing cancers provides a broader genomic profile and may point to tailored therapies and the need to screen at‐risk family members (Mandelker & Zhang, 2018).

Targeted multigene panels are providing definitive molecular diagnoses each year to hundreds of thousands of patients with clinically well‐defined disorders. CMA and whole‐exome sequencing (WES), on the contrary, provide unbiased assessments of the genome or exome, respectively, and are appropriate for patients with less well‐defined phenotypes or undiagnosed disease, particularly children with developmental delay. The diagnostic yields of CMA and WES are 15–20% and 25%,–40%, respectively (Miller et al., 2010; Wright, FitzPatrick, & Firth, 2018; Yang et al., 2014). WES is also useful for diagnosing certain phenotypes beyond nonspecific developmental abnormalities and has yielded positive results in a range of genetically heterogeneous immunological, skeletal, ophthalmological, and other disorders (Chou, Ohsumi, & Geha, 2012; de Castro‐Miro et al., 2016; Salvo et al., 2015; Stray‐Pedersen et al., 2017).

The growing use of NGS panels and WES has yielded three important outcomes worth highlighting. The first is the discovery of previously unrecognized clinical and genetic heterogeneity in several disorders and the refinement of genotype–phenotype correlations. As a good example of unrecognized allelic disorders, multigene NGS panel testing has shown that *KCNQ2* mutations commonly explain Ohtahara syndrome, an early infantile epileptic encephalopathy, whereas previously they were thought to be associated only with benign familial neonatal‐infantile seizures (Kato et al., 2013). As an example of underappreciated genetic heterogeneity, consider that breast cancer testing was largely restricted to *BRCA1* and *BRCA2* for almost three decades, but now at least 4% of positive molecular diagnoses are findings in other genes tested on NGS panels (Kurian et al., 2014)

A second key outcome of NGS and WES is the identification and classification of large numbers of variants from clinical and research testing and the discovery of rare private variants. Because tens to thousands of variants are discovered in individuals undergoing these tests, vast numbers of variants have been shared in public databases. For instance, ClinVar has 370,178 unique variants with interpretations at the time of this writing, and gnomAD has data from 123,136 exomes and 15,496 genomes. As a result, we now have a much more detailed picture of the prevalence of rare private polymorphisms in populations around the world that will come into even better focus as data from additional genomes are gathered and freely shared.

The third important outcome of NGS and, in particular, WES is the remarkable pace of novel disease gene discovery that has made the days of positional cloning seem charming (although those of us who cloned genes “in the dark” before the Human Genome Project had exciting adventures). More hereditary disorders have been discovered and characterized in the last 10 years than in the previous 30. Recent discoveries of novel disorders have occurred in studies of large clinical cohorts and through individual case studies connected through forums such as Matchmaker Exchange and GenomeConnect, which allow clinicians and researchers worldwide to communicate about novel genotypes and phenotypes and recognize patients with the same disorders (Kirkpatrick et al., 2015; Philippakis et al., 2015).

Alongside diagnostic genetic testing, proactive genetic screening is experiencing growth on three fronts: carrier screening, prenatal screening, and screening for adult‐onset disorders. Pan‐ethnic carrier screening is now a well‐established practice that has evolved from single‐variant detection in a select number of genes prescribed by professional guidelines to NGS‐based complete sequencing of genes for hundreds of autosomal recessive or X‐linked disorders. It is available globally to everyone interested in preconception screening. In countries with high rates of consanguineous marriage, WES has been proposed as a carrier screen (Sallevelt et al., 2017).

Prenatal genetic screening has largely transitioned from invasive amniotic fluid testing and chorionic villus sampling to noninvasive screening for aneuploidies, which is now expanding to include screening for specific microdeletion syndromes and even single‐gene disorders (Camunas‐Soler et al., 2018; Petersen et al., 2017). In certain cases, prenatal microarray testing and even WES are being offered in the absence of biochemical or ultrasound abnormalities to potentially identify known pathogenic variants associated with early‐onset and highly penetrant disorders. The American College of Obstetricians and Gynecologists does not support this routine use of prenatal CMA or WES for screening apparently normal pregnancies (Committee on Genetics & the Society for Maternal‐Fetal Medicine, 2016). However, as screening methods become noninvasive, more affordable, more technically robust, and easier to access through healthcare systems, the use of screens targeting increasing numbers of genetic loci will likely become routine worldwide.

Screening for variants related to medically actionable hereditary disorders is a recent development in proactive genetic screening that was prompted by the release of ACMG guidelines for reporting secondary findings from WES (ACMG Board of Directors, 2015). Although ACMG did not intend to propose that these genes be screened independent of a primary analysis of molecular etiologies for a presenting undiagnosed disease, some institutions have initiated programs to offer testing specifically of the genes listed in the guidelines to evaluate the clinical utility of such screening, its impact on health outcomes, and the potential economic implications (Carey et al., 2016; Roche & Berg, 2015). Integrated healthcare systems are beginning to incorporate this screening in routine healthcare (Carey et al., 2016). This trend is further bolstered by advocates of routine screening for *BRCA1* and *BRCA2* variants in all adult women (King, Levy‐Lahad, & Lahad, 2014). Emerging evidence suggests that 2%,–5% of individuals in the general population carry pathogenic variants in the 59 ACMG‐listed genes for clinically actionable disorders (Dorschner et al., 2013; Gambin et al., 2015; Olfson et al., 2015). Identifying these individuals is a first step, but accurately determining their risk for manifesting a disorder remains a considerable challenge because there are very few long‐term studies of such individuals that address the rates of penetrance beyond certain genotypes. Nevertheless, as the cost of these types of screens declines and evidence of their clinical utility becomes richer, more clinicians and their patients will likely demand them as part of overall health management. Indeed, an elegant study recently showed that Mendelian disease prevalence may be underappreciated and can even overlap phenotypes seen in complex disease (Bastarache et al., 2018). Ultimately, we expect that whole‐genome sequencing will be performed as a screening modality beginning in childhood so that applicable and relevant genetic information can be summarized in medical records and information relevant to disease risk, therapeutics, and family planning can be delivered on demand throughout an individual's lifespan.

Compared with numbers even five or 10 ten years ago, many more people around the world are now having their genomic information analyzed. We are therefore witnessing a relatively new phenomenon in medicine: a self‐reinforcing free flow of information among nodes in a modern genomics ecosystem aimed at improving clinical interpretation, establishing diagnoses, supporting research and discovery, and connecting individuals to effective therapies (Figure 3). In addition to important initiatives such as ClinVar, gnomAD, DECIPHER, the Database of Genomic Variants, HGMD, and LOVD, new genomic data projects such as Genome England, the Arab Genome Project, and the All of Us Research Program at the U.S. National Institutes of Health (https://allofus.nih.gov/) are emerging to provide deeper insight into variation in the human genome, in turn improving the interpretation of variants (Al‐Ali, Osman, Tay, & AlSafar, 2018; Saudi Genome Project Team, 2015; Turnbull, 2018). However, these and future enormous data sets will increasingly demand AI tools to support research and reach medically important conclusions. The digitization of genomic information lends itself to AI‐based analysis, and promising platforms such as Google's DeepMind and IBM Watson are reaching into genomics (https://www.ibm.com/watson/health/oncology-and-genomics/genomics/). Additional platforms will be developed during the next decade, essentially making AI tools standard requirements for genomics analysis. Clinical apps using facial recognition technology are also being developed to support facial dysmorphology assessments in pediatric disorders (Kruszka et al., 2017; Liehr et al., 2018). Aside from aiding clinical diagnoses, AI will enhance the discovery of novel disease genes, refinement of genotype–phenotype correlations, characterization of penetrance, and dissection of complex multifactorial genetic conditions.

**Figure 3:**
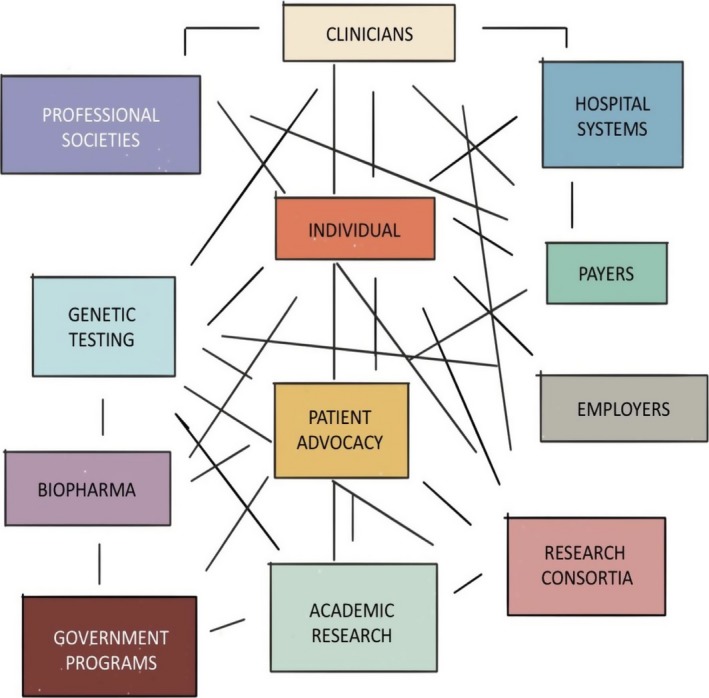
The modern genomics ecosystem. We are seeing burgeoning interactions and influence among a number of stakeholders who generate or consume genomic information to manage health, perform research, establish medical practice guidelines, establish third‐party reimbursement criteria for genetic tests, enact government‐mandated programs (e.g., newborn screening), develop novel therapies, and assemble groups of patients with the same genetic disorders to promote advocacy and support. Integrated hospital systems are exploring the routine use of genomics for their patients. More recently, some large employers have offered genetic screening for their workforce to promote proactive health management. Even life insurance companies are now exploring proactive genetic screening in an effort to prolong the lives of their subscribers

A particularly critical node in the flourishing genomics ecosystem is rare disease therapy (Sun, Zheng, & Simeonov, 2017). The results of diagnostic testing can have significant clinical management implications for patients with certain genetic disorders. Many of these are biochemical disorders, but others include certain cancer and cardiovascular syndromes. Interest is increasing in the development of new therapies for rare diseases, which affect more than 25 million individuals in the U.S. and more than 300 million worldwide (https://rarediseases.info.nih.gov). The last few years have witnessed the successful development of life‐altering rare disease therapies (Table 1), and as the costs of genetic testing decline, more affected individuals will be able to receive molecular diagnoses that qualify them for therapies available on the market or through clinical trials. Closer partnerships among biopharma, genetic testing providers, and academic researchers are benefiting patients with rare disease like never before.

**Table 1:** Examples of genetic disorders for which therapies have been developed and genetic testing is available for or included as part of the clinical diagnostic process

Disorder	Therapy	Company
CLN2 neuronal ceroid lipofuscinosis (classic late infantile Batten disease)	Enzyme replacement therapy (Brineura)	BioMarin
Hereditary TTR amyloidosis	RNAi therapeutic (Patisiran)	Alnylam
X‐linked hypophosphatemic rickets	Monoclonal antibody (Crysvita)	Ultragenyx
Leber congenital amaurosis	Gene therapy (Luxturna)	Spark Therapeutics
Hypophosphatasia	Enzyme replacement therapy (Strensiq)	Alexion
Periodic paralysis	Carbonic anhydrase inhibitor (Keveyis)	Strongbridge

In conclusion, a modern ecosystem for clinical genomics has emerged because of the rapid evolution and consolidation of high‐throughput, high‐resolution technologies and bioinformatics solutions, the creation of vast databases of genomic information from populations around the world, the accelerating discovery of novel disease genes and genotype–phenotype correlations, and the evolution of networks that disseminate genomic information among diverse stakeholders. Catalyzing this progress is the rapidly decreasing cost of DNA sequencing and availability of multi‐gene panels, which have provided ready access to high‐quality genetic testing worldwide. This deeper integration of genetics in mainstream medicine will influence healthcare decisions across patient lifespans, drive personalized approaches to precision medicine, and help connect individuals to others with shared genotypes and phenotypes.

All of these advances portend a bright future for genomics‐enabled medicine, although formidable challenges remain and should not be underestimated. We need to improve the accuracy of penetrance rates for all disorders, recognize the influence of population‐specific genomic backgrounds, aggregate useful phenotype data to uncover meaningful genotype–phenotype correlations, collect health outcomes data in both diagnostic and preventive genetics, and of course, develop safeguards for privacy of and access to genomic information. Medical education around the world should also provide more exposure to genetics. Even when these knowledge gaps in clinical genomics are filled and the challenges to integrating genomics into mainstream medicine are overcome, serious and daunting economic issues will remain due to healthcare inequities across the globe. Per capita annual healthcare expenditures range from more than $9,600 in developed countries in Western Europe to less than $20 in some countries in Africa and Southeast Asia (WHO Global Health Expenditure Database; http://apps.who.int/nha/database/Select/Indicators/en). Genomics will need to demonstrate its value if it is to have its anticipated impact on global healthcare. Nonetheless, with the momentum of the twin forces of the falling cost of testing and the expanding knowledge of how to interpret the impact of genetic information on health, we anticipate that these challenges will be overcome. We are now witnessing the start of this exciting future.

## CONFLICT OF INTEREST

The authors are medical team leaders and executives at Invitae, a genetic testing laboratory.
